# Cost-effectiveness of a Novel Hypoglycaemia Programme: The ‘HARPdoc vs BGAT’ RCT

**DOI:** 10.1111/dme.15304

**Published:** 2024-02-29

**Authors:** Andrew Healey, Tayana Soukup, Nick Sevdalis, Ioannis Bakolis, Samantha Cross, Simon R. Heller, Augustin Brooks, Dulmini Kariyawasam, Elena Toschi, Linda Gonder-Frederick, Marietta Stadler, Helen Rogers, Kimberley Goldsmith, Pratik Choudhary, Nicole de Zoysa, Stephanie A. Amiel, for the HARPdoc RCT Study Group

**Affiliations:** 1 Centre for Implementation Science, Health Service and Population Research Department, Institute of Psychiatry, Psychology and Neuroscience, King’s College London, London, UK; 2 Department of Biostatistics and Health Informatics, Institute of Psychiatry, Psychology and Neuroscience, King’s College London, London, UK; 3 Department of Oncology and Metabolism, University of Sheffield, Sheffield, UK; 4 University Hospitals Dorset NHS Foundation Trust, Bournemouth, UK; 5 Department of Diabetes and Endocrinology, Guy’s and St Thomas’ NHS Foundation Trust, London, UK; 6 Guy’s and St Thomas’ NHS Foundation Trust, London, UK; 7 Joslin Diabetes Center, Harvard Medical School, Boston, Massachusetts, USA; 8 Department of Psychiatry and Neurobehavioral Sciences, University of Virginia, Charlottesville, USA; 9 Department of Diabetes, School of Cardiovascular and Metabolic Medicine & Sciences, King’s College, London, UK; 10 Department of Diabetes, King’s College Hospital NHS Foundation Trust, London, UK; 11 Leicester Diabetes Centre, University of Leicester, Leicester, UK

**Keywords:** cost-effectiveness, health economics, hypoglycaemia, quality of life, randomised controlled trial, service utilization, type 1 diabetes

## Abstract

**Aims:**

To assess the cost-effectiveness of HARPdoc (Hypoglycaemia Awareness Restoration Programme for adults with type 1 diabetes and problematic hypoglycaemia despite optimised care), focussed upon cognitions and motivation, versus BGAT (Blood Glucose Awareness Training), focussed on behaviours and education, as adjunctive treatments for treatment-resistant problematic hypoglycaemia in type 1 diabetes, in a randomised controlled trial.

**Methods:**

Eligible adults were randomised to either intervention. Quality of life (QoL, measured using EQ-5D-5L); cost of utilisation of health services (using the adult services utilization schedule, AD-SUS) and of programme implementation and curriculum delivery were measured. A cost-utility analysis was undertaken using quality-adjusted life years (QALYs) as a measure of trial participant outcome and cost-effectiveness was evaluated with reference to the incremental net benefit (INB) of HARPdoc compared to BGAT.

**Results:**

Over 24 months mean total cost per participant was £194 lower for HARPdoc compared to BGAT (95% CI: −£2498 to £1942). HARPdoc was associated with a mean incremental gain of 0.067 QALYs/participant over 24 months post-randomisation: an equivalent gain of 24 days in full health. The mean INB of HARPdoc compared to BGAT over 24 months was positive: £1521/participant, indicating comparative cost-effectiveness, with an 85% probability of correctly inferring an INB > 0.

**Conclusions:**

Addressing health cognitions in people with treatment-resistant hypoglycaemia achieved cost-effectiveness compared to an alternative approach through improved QoL and reduced need for medical services, including hospital admissions. Compared to BGAT, HARPdoc offers a cost-effective adjunct to educational and technological solutions for problematic hypoglycaemia.

## 1 | INTRODUCTION

Despite advances in blood glucose monitoring and insulin delivery, hypoglycaemia (low blood glucose) continues to complicate insulin therapy for type 1 diabetes (T1D).^1^ Although continuous glucose monitoring (CGM) and other diabetes technologies significantly reduce severe hypoglycaemia (SH, episodes in which a person requires treatment by another because of cognitive impairment^2^), residual risk remains in up to 20% of people using such systems.^1^ The technologies have not been shown consistently to restore impaired awareness of hypoglycaemia (IAH), a major risk factor for SH.^1,3^ SH can result in confusion, coma, cardiovascular events and, rarely, death.^4^ Although most episodes are treated by family, friends and members of the public, some result in ambulance call-out and hospital admission, with associated costs.^5,6^ IAH increases the risk of SH in T1D six-fold.^3^ It is associated with reduced mental health in people with IAH^7,8^ and family members^9^ and may incur indirect health and social cost.^6^ IAH plus recurrent SH has been termed “problematic hypoglycaemia”.^10^ Currently we have only cell replacement therapy by islet transplantation to offer to people whose problems persist after structured education in flexible insulin therapy and technologies. Transplantation is limited by graft durability, donor availability, fitness for surgery and risks of long-term immunosuppression, making it inappropriate or unacceptable for many. The role of re-education in this setting has not been evaluated and there is no other evidence-based therapy to offer.^10^

The Hypoglycaemia Awareness Restoration Programme for adults with T1D and problematic hypoglycaemia persisting despite optimised care (HARPdoc) is a novel psycho-educational intervention which uniquely uses psychological theory to help people address unhelpful thoughts around hypoglycaemia.^11^ It is based on evidence that people with severe IAH endorse thoughts about hypoglycaemia that act as barriers to their ability to gain benefit from interventions that should reduce hypoglycaemia and restore awareness.^12,13^ HARPdoc is delivered by two diabetes educators, trained and supported by a clinical psychologist, who facilitate sessions for up to eight participants, over 6 weeks, with full-day attendance on 4 days and 2 one-to-one telephone sessions.^11^ Family members join week 6 and there are group follow-ups at 3 and 6 months. In a recent randomised controlled trial (RCT), HARPdoc was tested against the psycho-educational programme, Blood Glucose Awareness Training (BGAT). BGAT teaches about internal and external associations of high and low blood glucose and how best to avoid both, without the cognitive and motivational elements that characterise HARPdoc and is generally delivered in eight 2-h sessions by one diabetes educator.^14^ The National Institute for Health and Care Excellence, NICE, recommends BGAT for problematic hypoglycaemia,^15^ although the original evidence-based BGAT curriculum is not currently available.^16^ In the RCT, HARPdoc reduced SH and improved awareness scores no more than did BGAT but was associated with improved mental health scores, which BGAT was not.^17^

Given the potential importance of the mental health gains seen with HARPdoc in a highly vulnerable group of people with T1D and the need for a solution for their problematic hypoglycaemia, we report a within-trial economic evaluation of HARPdoc, with BGAT as its comparator.

## 2 | METHODS

### 2.1 | Study design

This was an incremental cost-utility analysis (CUA) of HARPdoc with BGAT as comparator.^18^ The analysis was undertaken from a UK National Health Service (NHS) and social care services perspective. This pre-planned analysis draws on intervention implementation and delivery data, patient self-reported health service use and quality-of-life data collected from UK participants during the randomised parallel two-arm trial that ran from March 2017 to July 2021. The RCT’s primary outcome was rate of SHs (number over preceding year) between groups at 12- and 24-month follow-up. Study design,^11,19^ primary and secondary outcomes and main clinical effectiveness results have been reported.^17^

### 2.2 | Population

Adults (≥18 years) with T1D for ≥4 years, IAH (Gold and/or Clarke score ≥4 [8, 29]) and >1 episode of SH in the previous 2 years, at least one on current treatment modality, were eligible. Participants were required to have previously attended structured education in flexible insulin therapy and be under active care of a specialist diabetes centre with access to diabetes technologies (CGM and CSII).^11^

### 2.3 | Study setting

This economic evaluation is restricted to UK study participants (from London (King’s College Hospital and Guy’s and St Thomas’s NHS Foundation Trusts); Sheffield Teaching Hospitals NHS Foundation Trust; and the Royal Bournemouth Hospital), who formed 83% of those recruited.

### 2.4 | HARPdoc and BGAT comparator

HARPdoc courses comprised one full-day group meeting in weeks 1–3 and 6, with sessions also for relatives in week 6. Morning sessions lasted up to 4 h, and afternoon sessions 3 h. Two diabetes educators, a specialist nurse and a dietitian, were trained and supported to deliver sessions by a clinical psychologist, leading the meetings, with a consultant physician attending parts of meetings in weeks 1 and 6. Scheduled one-to-one sessions between educators and participants were held in weeks 4 and 5, with optional unscheduled contacts if required. BGAT was updated and configured to run over the same time frame but was less resource intensive: group meetings were shorter (scheduled as two-hour sessions morning and afternoon), with one diabetes educator.

## 3 | TRIAL DATA FOR ECONOMIC E VA LUATION

### 3.1 | Health care utilisation

Participant health care and other service utilisation over follow-up were measured using an adapted self-report Adult Service Utilisation Schedule (AD-SUS)^20^ administered 12 and 24 months post-randomisation by research staff by telephone or face-to-to-face. Respondents were asked to report frequency of contact with a range of hospital and community-based services during the previous 12 months (Table S1).

### 3.2 | Intervention resource inputs

Interviews with all health care professionals (*n* = 28, including 7 from our US centre) involved with course delivery in the RCT were undertaken to determine, retrospectively, time allocated to delivery activities of each programme, including administrative and group session preparatory time; receipt of training and supervision of educators; time spent by the clinical psychologists delivering training and supervision and travel time required to deliver group courses (including travel expenditures).^19^ We refer to these as ‘implementation costs’.^21,22^

Trial participant attendance at group meetings and frequency of scheduled and unscheduled one-to-one contacts were recorded by protocol at each study site. These comprise ‘curriculum costs’, which thus include the costs of delivering the intervention.

### 3.3 | Unit costs

Published unit cost estimates for health and social care professionals and services^23^ and NHS reference costs (https://www.england.nhs.uk/costing-in-the-nhs/national-cost-collection/#ncc1819) were used to cost self-reported contact with health care professionals. Costs are reported at 2020/2021 prices (Table S2).

### 3.4 | Calculation of trial participant costs

Health care utilisation per participant was costed by multiplying the number of self-reported ‘units’ of service or care professional contacts per individual by its unit cost. Implementation costs were estimated as the total volume of time allocated to each activity multiplied by the hourly employment cost for each relevant health care professional.^22^

Total implementation costs for each programme were divided by the number of participants randomised to that trial arm, giving an implementation cost per participant. Curriculum costs for each scheduled group meeting were calculated as its total anticipated running time multiplied by the hourly cost for each health care professional in attendance. The cost per participant for each group meeting was estimated as the total cost for each scheduled group meeting divided by the recorded number of attendance during a specific week. Cost of one-to-one contacts was estimated as the sum of contacts for each participant multiplied by the unit cost for the relevant health care professional (assuming 30 min/contact). Total curriculum costs were calculated as total group meeting cost per participant added to the cost of one-to-one contacts.

The cost-utility analysis used the total cost per participant in each trial arm:

Total cost over 2-year follow-up (per participant) = total cost of health service utilisation per participant + total implementation cost per participant+ total curriculum cost per participant.

Costs were calculated in pounds sterling and are presented at 2020–21 price levels.

### 3.5 | Outcome measurement

Health-related quality of life outcomes was measured by participants using the EQ-5D-5L^24^ at baseline, 12 and 24 months. Participants reported across five domains (mobility, self-care, usual activities, pain, and anxiety/depression), rating current impairment e4perienced (‘none’ to ‘extreme’). From these, each participant was assigned to one of 3125 unique health states. Following recent NICE guidelines,^25^ we applied health states applicable to the 3-level version of EQ-5D that were derived using a ‘cross-walking’ algorithm developed by Van Hout and colleagues to the 5-level EQ-5D health state data.^26^ ‘Utility’ weights applicable to the 3-level version were then used to estimate quality-adjusted life years (QALYs) for each participant from baseline to 12 months and from 12 to 24 months using the area-under-the-curve method.^27^ The utility weights reflect UK-specific community preferences over different states of health, ranging from a maximum value of 1 (corresponding to full health) to 0 (death), with negative values allowed for states considered worse than being dead.^28^ Cross values were used. The time horizon over which QALYs and costs were measured covered a 24-month period from randomisation split into two 12-month periods. This time period was selected as it corresponded to the period of evaluation for the main clinical trial. Costs and QALYs 12- to 24-month period were conventionally discounted at a rate of 3.5%, following standard practice.^29^

## 4 | ANALYTICAL APPROACH

A ‘net benefit’ approach was adopted to determine the cost-effectiveness of HARPdoc vs BGAT. We chose this method as it offers a single intuitive summary measure of programme cost-effectiveness that can be estimated from the trial data in single step when an assumption is made regarding how much a resource-constrained health system should be prepared to pay to gain a single QALY (the cost-effectiveness threshold).^30^ The incremental net benefit (INB) of HARPdoc vs BGAT was defined as:

$$\text{INB=}\left(\Delta Q\times \lambda \right)-\Delta C$$

ΔQis the difference in average (mean) QALYs over 24 months between trial arms (the incremental health benefit of HARPdoc); ΔCis the difference in mean total cost (incremental cost). λis the cost-effectiveness threshold referred to above. NICE currently set this threshold at £20,000 to £30,000. If the INB > 0 an intervention can be considered a cost-effective substitute for its comparator.

### 4.1 | Statistical methods

To account for clustering at group level (in both arms), a two-level linear mixed-effects random intercepts model was fitted to estimate the difference in QALYs between trial arms and the difference in mean total costs at each follow-up point separately. All models included a trial allocation dummy variable to facilitate identification of QALY and cost differences. Additional covariates added to the model included: baseline EQ-5D-5L transformed utility scores (QALY model only); a fixed-effect for trial stratification (use of insulin pumps and/or CGM vs multiple daily injections with finger-prick monitoring); baseline covariates predictive of missing cost and QALY outcomes for randomised participants; and additional covariates whose inclusion/exclusion had important implications for the magnitude of estimated cost and QALY differences (identified through stepwise deletion). For handling missing outcomes data, our analysis was carried out on an available case basis and assumed that outcomes are CD-MCAR (missing completely at random conditional on baseline covariates predictive of missingness).^31^ Exploration of outcomes data over follow-up found no support for adoption of an alternative missing at random (MAR) assumption and the application of imputation methods built on MAR.

To factor in uncertainty caused by trial sampling error, mixed-effects cost and QALY models were bootstrapped 1000 times to generate a plausible joint density for estimated differences in mean cost and QALY outcomes. A unique INB value corresponding to *λ* = £20,000, the lower bound of the NICE range, was calculated for each joint pairing of cost and QALY differences. The mean INB from the distribution (a ‘best estimate’ of its value given sampling uncertainty) was used to evaluate whether HARPdoc is cost-effective compared to BGAT. We also present the probability of HARPdoc being the cost-effective alternative by consideration the proportion of bootstrap distribution yielding an INB > 0.

### 4.2 | Sensitivity analysis

The sensitivity of cost-effectiveness conclusions to differences in the assumed value of *λ* were examined: repeating the above analysis assuming *λ* = £30,000 per QALY gained (NICE’s upper bound); and *λ* = £13,000, a value approximating a recently recommended cost-effectiveness threshold value argued to embody a more realistic assessment of the opportunity cost of additional expenditure on new health programmes in the NHS.

We also tested the robustness of ‘base case’ conclusions to: exclusion of costs relating to non-elective bed-day utilisation the principle mechanism of impact on inpatient admissions was expected to be through the avoidance of emergency admissions relating to the primary outcome (SH events); adjusting for the presence of a very high-cost non-elective admission in the HARPdoc trial arm (setting it to the mean cost of other non-elective admissions in the trial sample); and adjustment for a high-cost psychiatric admission in the BGAT arm (setting this cost to zero).

Analyses were performed in Stata (version 17). Reporting follows CHEERS guidelines,^32^ with reference to a Health Economics Analysis Plan (HEAP).

## 5 | RESULTS

Of the 82 UK participants included, 41 were randomised to each intervention. Of those randomised, 58 (71%) undertook the AD-SUS at 12 and 65 (79%) at 24 months. EQ-5D-5L was administered to 71 (87%) and 63 (77%) randomised participants at 12 and 24 months respectively. Component data were completed for all EQ-5D questionnaires over follow-up. A small number of health care professionals interviewed had incomplete data relating to single items of service contact required for estimating total costs (3 at 12 months; 6 at 24 months).

Table 1 describes mean intervention costs per trial participant.

Cost of implementation and the course delivery was estimated as higher for HARPdoc: total intervention costs (implementation plus course curriculum costs) being £1697 per participant for HARPdoc, and £541 for BGAT.

Table 2 shows the AD-SUS data. There were fewer contacts with community-based services, accident and emergency episodes and hospital admissions at both 12 and 24 months in HARPdoc participants, and fewer outpatient contacts at 12 months. Mental-health-related contacts (psychiatrist, psychologist and counselling) were lower at 12 and 24 months after HARPdoc as were diabetes clinic visits at 24 months. Routine scheduled visits, such as with opticians, were not obviously different between groups.

**FIGURE 1:**
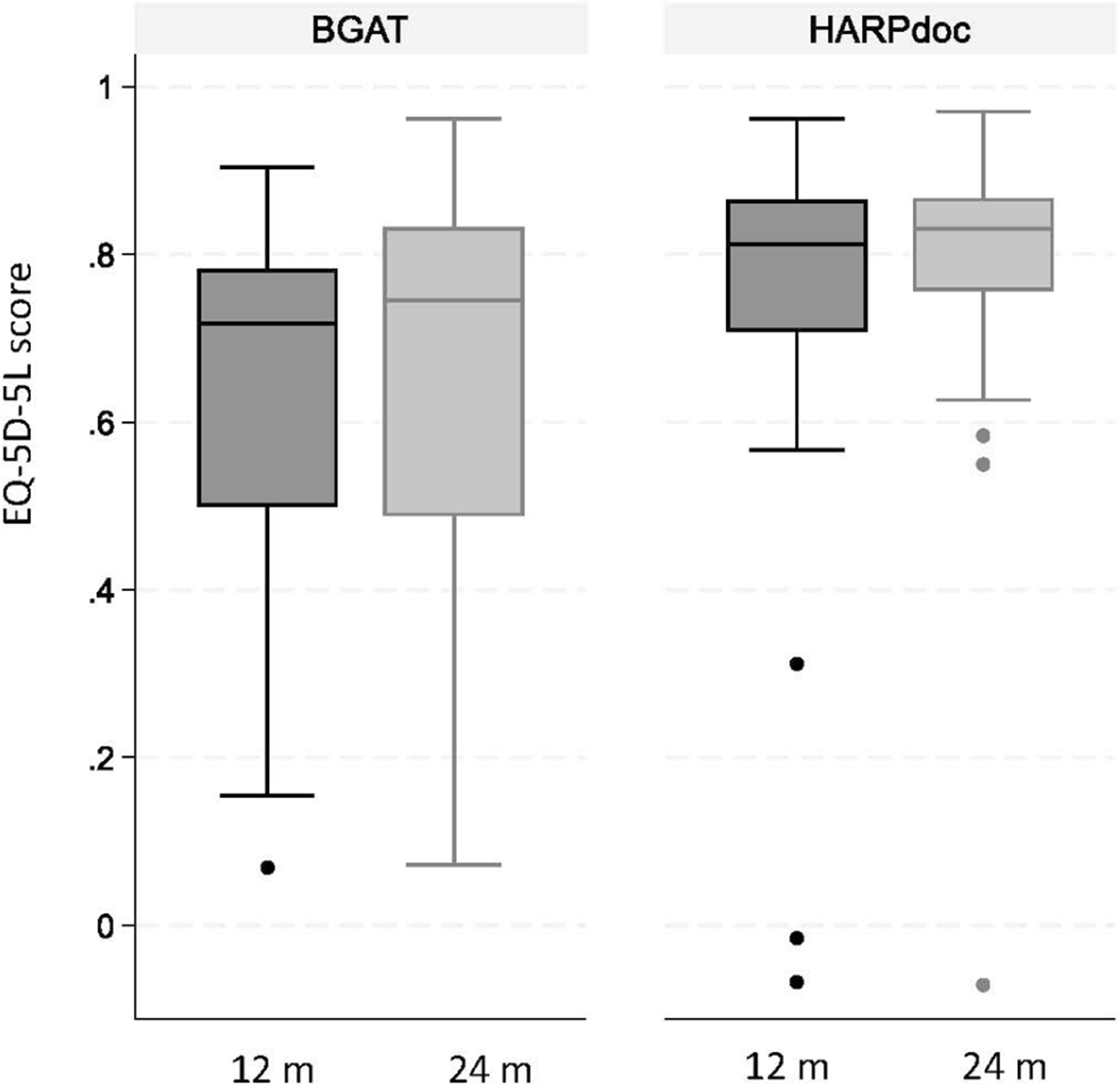
Box plot of linear predicted values for EQ5D-3L scores by trial arm adjusting for baseline scores. Predicted scores obtained from linear (OLS) regression of 12- and 24-month EQ5D-3L scores with trial arm and baseline EQ5D-3L scores included as covariates. Dark bars represent linear predicted score at 12 months, light bars represent linear predicted score at 24 months, each showing 25–75 percentile, with horizontal lines representing median score, whiskers maxima and minima and closed circles outliers.

Figure 1 presents a box plot describing the distribution of predicted EQ5D-3L utility scores at 12 and 24 months for each trial arms adjusting for baseline EQ5D-3L utility values, showing higher values for HARPdoc at both 12 and 24 months. Table 3 presents the main cost-effectiveness results. The expected total cost was £525 higher (95% CI: −£832 to £1735) for each HARPdoc than BGAT participants between baseline and 12 months; and £719 lower (95% CI: −£2585 to £895) between 12 and 24 months. The expected total mean cost was £194 lower (95% CI: −£2498 to £1942) over the 24-month follow-up, HARPdoc vs BGAT. HARPdoc participants were expected to have accumulated a mean of 0.067 more QALYs per participant over 24 months (95% CI: −0.024 to 0.155) than BGAT participants (Table 1): equivalent to a gain of around 24 days spent in full health over 2 years.

Combining incremental cost and health benefit evidence, HARPdoc was a cost-effective alternative to BGAT with a positive mean INB of £1521 over 24 months, assuming a cost-effectiveness threshold of £20,000 per QALY gained. This was robust to the application of a more stringent threshold (£13,000) per QALY gained. After accounting for trial sampling error, the probability that HARPdoc was a cost-effective alternative to BGAT varied between 80% and 89% across the chosen thresholds. These results are also summarised visually as a cost-effectiveness acceptability curve (CEAC, Figure S1). The core conclusions regarding the comparative cost-effectiveness of HARPdoc based on mean INB values were robust to all additional sensitivity analyses (exclusion of non-elective bed day costs, adjustment for extreme bed days cost; and omission of psychiatric inpatient cost).

## 6 | DISCUSSION

The present data show that, at least in the UK healthcare context, the HARPdoc intervention for adults with T1D and problematic hypoglycaemia persisting despite otherwise optimised diabetes care was a more cost-effective alternative to the comparator intervention, BGAT, in a randomised evaluation. This superiority was driven by reduced use of health care services by HARPdoc participants and a gain in quality of life, across 2 years. Although reductions in the cost of wider service utilisation were here largely driven by fewer hospital admissions for HARPdoc trial participants, cost of other types of service contacts were also lower for them. These differences offset the relatively high cost of implementing and delivering the HARPdoc programme, resulting in HARPdoc being the more cost-effective intervention. While sampling error means that there was some margin for error around our conclusions, the probability that HARPdoc was superior to BGAT in terms of comparative cost-effectiveness was 85% at a cost-effectiveness threshold of £20,000.

Our analysis was not powered to detect statistically significant differences in total cost or the cost of specific types of service contact. However, at a descriptive level, the pattern of reduced use of other health services by HARPdoc participants is of interest. There were fewer visits to primary care physicians and nurses (a median of <3 vs. >4, HARPdoc vs BGAT) across the 24-month follow-up, and reduced visits to diabetes services in the second 12 months, suggesting greater confidence in self-management of diabetes and perhaps other health issues. The lack of an obvious difference in contact with screening services such as eye tests adds validity to the findings. The described reduced use of mental health services in HARPdoc participants is consistent with improved mental health scores (diabetes distress, anxiety and depression) reported in the trial main outcomes.^17^ AD-SUS data were not collected at baseline, but participants did complete a questionnaire including items about contact with psychological therapies, with no evidence of baseline between-group differences.^17^ Given high rates of diabetes distress, anxiety and depression in people with IAH,^7,8^ these findings have potential clinical importance. Hospital admissions, lower in the HARPdoc follow-up, were infrequent overall. However, cost-effectiveness was maintained after exclusion of cost differences relating to non-elective admissions.

HARPdoc was designed to address a problem experienced by a specific group of people, rendered highly vulnerable by hypoglycaemia risk persisting despite other evidence-based interventions. In a recent analysis from the US T1D Clinic Exchange, nearly 20% of those using CGM and over 15% using hybrid closed loop continued to report SH,^1^ IAH remaining a strong association.^3^ We speculate that cognitive barriers to hypoglycaemia avoidance may be a significant contributor to residual problematic hypoglycaemia with current technology, and account for the persistence of the phenomenon of about 10% of people accounting for most reported SH. It is these cognitions that HARPdoc addresses. All HARPdoc RCT participants were attending specialist diabetes services, offering structured education (a requirement for participation) and access to technologies, particularly insulin pump therapy and/or CGM, with a mandate in the UK to be offered to those with hypoglycaemia.^33,34^ About 80% of participants had been offered pump and/or CGM, and although many had tried it, only 50% were using technology at baseline.^35^ This supports the hypothesis that unaddressed cognitive barriers may prevent successful engagement with conventionally offered support in an otherwise motivated group. HARPdoc should be seen as an adjunct to, not a replacement for, education, CGM and hybrid closed loop, working to enhance their efficacy, although evidence from the trial suggests it works independently of technology use too. Consequently, it may also provide an alternative, after education and ideally also after at least CGM. The superior QALY/EQ5D values for HARPdoc reported here are very consistent with improvements in mental health seen with the programme in the clinical outcomes analysis. Anxiety and depression are domains within the EQ5D and we reported improvement in scores for diabetes distress, non-specific anxiety and depression (high in the whole trial cohort at baseline^35^) was seen only in the HARPdoc group in that analysis.^17^ We hypothesised that the motivational facilitation style in HARPdoc, designed to increase self-efficacy and empower participants to address problems around hypoglycaemia, has had a ‘spill-over’ effect that has helped them cope better with other issues, perhaps contributing to their lesser requirement for health services in general. HARPdoc also addressed styles of thinking such as catastrophising and perfectionism applied to blood glucose management, that are associated with anxiety and depression. We now suggest that the improvement in mental health, unique to the HARPdoc programme, is driving the improved quality of life, detected here by the EQ-5D, a generic measure which is not tailored for diabetes or hypoglycaemia.

Limitations of our study include its relatively small size and that the cost-effectiveness analysis has been carried out on data from a randomised controlled trial carried out in a specific geographical context. The latter has benefits, in that it was a pre-planned implementation science investigation of the RCT, with robust data collection, but although our use of micro-costing allows us to take a ‘real-life’, hence scalable, approach to what it may cost to deliver these interventions outside a trial setting, the findings will need testing in a service delivery model. Our data are specific to the UK’s NHS, although they provide evidence that will be informative to other contexts. We compared HARPdoc with BGAT, and our findings should not be taken as an indication of the cost-effectiveness of HARPdoc compared to usual care in the NHS, or elsewhere, without existing provision for this patient group. It should also be noted that the costs for implementing HARPdoc may have been exaggerated by inclusion of all of the training of the educators for just the participants of the trial – once trained, educators can continue to deliver courses over more extended periods to more patients without need for retraining, although on-going clinical psychology supervision is important. Service utilization costs depended on participant recall, albeit using an established questionnaire administered by researchers, with no difference in method between interventions.

People who continue to experience IAH and recurrent SH despite best efforts with conventional management need more support. The physical and mental health burden they experience is just beginning to be explored,^7,8^ but there are data showing increased use of health services following hypoglycaemia,^36^ which adds costs to those of the acute management, which are considerable, notwithstanding that most SH is managed by family and friends. It is important to recognise that the cost-effectiveness of HARPdoc has been demonstrated against another intervention, not against usual care, as participants were experiencing their problematic hypoglycaemia despite access to the existing usual care pathway for their problem. To provide context, a conservative estimate of the reduction in cost for SH-associated ambulance call out, ER attendance and hospital admissions, seen with trial arms over the 2-year trial was £30,471 per 100 patients per year, equating to savings ranging between £52 and £131 million per year in the UK (see Table S3 for assumptions made). This cost saving might be made with either intervention but HARPdoc additionally provides improvement in mental health outcomes^17^and the present analysis shows associated quality-of-life improvements and reduced need for health services including admissions. We conclude that HARPdoc offers a cost-effective alternative to the recommended programme (BGAT) for people with diabetes within the NHS and, by extension, other integrated health service systems.

## Supplementary Material

supplemental material

Additional supporting information can be found online in the Supporting Information section at the end of this article.

## Figures and Tables

**TABLE 1:** Intervention cost per trial participant.

	HARPdoc: mean (SD)	BGAT: mean (SD)
Implementation cost^a^ (HCP time)		
Receipt of training	£333	£146
Delivery of training	£187	£60
Course preparatory work	£210	£84
Travel time and expenditure	£150	£70
Curriculum cost (HCP time)		
Group meetings	£743 (£290)	£168 (£76)
One-to-one contacts (scheduled)	£54 (£15)	£11 (£23)
One-to-one contacts (unscheduled)	£14 (£40)	£6 (£28)
Total intervention cost	£1697 (£290)	£541 (£82)

Abbreviations: HCP, health care professional; SD, standard deviation.

a Estimated from interviews with staff involved with implementation and delivery. Total implementation costs divided through by number of participants randomised. Therefore, sample variation (standard deviation) not estimable.

**TABLE 2:** Utilisation of health services during course of first and second 12-month periods of the trial, HARPdoc and BGAT.

	HARPdoc				
	Baseline to 12 months *n* = 33			12 to 24 months *n* = 33	
	Mean	SD	Min-Max	Mean	SD
(A) Community-based services					
General practitioner	£72.37 (1.97 contacts)	£83.48	£0 to £256.46	£38.83 (1.15 contacts)	£54.21
Primary care nurse	£13.94 (0.73 contacts)	£18.73	£0 to £95.9	£17.87 (1.09 contacts)	£20.37
Podiatrist	£26.51 (0.45 contacts)	£72.37	£0 to £336.96	£63.66 (1.15 contacts)	£190.67
Optician	£13.93 (0.39 contacts)	£19.65	£0 to £70.74	£11.79 (0.33 contacts)	£21.05
Pharmacist	£2.76 (0.36 contacts)	£6.72	£0 to £25.20	£2.04 (0.24 contacts)	£4.71
District nurse	£0.58 (0.03 contacts)	£3.34	£0 to £19.18	£0.00 (0.00 contacts)	_
Dietician	£1.26 (0.06 contacts)	£5.04	£0 to £20.78	£0.00 (0.00 contacts)	–
Physiotherapist	£5.75 (0.09 contacts)	£24.31	£0 to £126.48	£11.97 (0.24 contacts)	£45.41
Occupational therapist	£2.26 (0.03 contacts)	£12.96	£0 to £74.46	£0.00 (0.00 contacts)	–
Psychiatrist	£7.63 (0.03 contacts)	£43.80	£0 to £251.64	£7.63 (0.03 contacts)	£43.80
Psychologist	£15.13 (0.15 contacts)	£86.90	£0 to £499.20	£0.00 (0.00 contacts)	–
Psychotherapist	£0.00 (0.00 contacts)	–	–	£0.00 (0.00 contacts)	–
Counsellor	£0.00 (0.00 contacts)	–	–	£0.00 (0.00 contacts)	–
Social worker	£1.58 (0.03 contacts)	£9.06	£0 to £52.02	£0.00 (0.00 contacts)	–
Home help	£289.31 (9.45 contacts)	£1223.17	£0 to £6364.80	£48.22 (1.58 contacts)	£276.99
Meals on wheels	£0.00 (0.00 contacts)	–	–	£0.00 (0.00 contacts)	–
Other community	£6.17	£35.42	£0 to £203.49	£79.73	£386.81
Total cost of community-based service contacts	£457.60	£1269.70	£0 to £6664.20	£281.73	£523.87
(B) Secondary care contacts Outpatients					
Diabetes clinic	£490.28 (3.12 contacts)	£497.91	£0 to £2199.21	£342.72 (2.18 contacts)	£371.42
Foot clinic	£119.00 (0.76 contacts)	£492.10	£0 to £2827.44	£38.08 (0.24 contacts)	£96.43
Eye clinic	£176.12 (1.12 contacts)	£332.66	£0 to £1884.96	£114.24 (0.73 contacts)	£143.07
Ophthalmology	£63.43 (0.58 contacts)	£120.14	£0 to £440.64	£53.41 (0.48 contacts)	£110.58
Dietetics	£8.35 (0.09 contacts)	£35.28	£0 to £183.60	£11.13 (0.12 contacts)	£44.49
General outpatients	£60.52 (0.33 contacts)	£185.30	£0 to £726.24	£71.52 (0.39 contacts)	£163.27
Day surgery	£25.13 (0.03 contacts)	£144.36	£0 to £829.26	£100.52 (0.12 contacts)	£274.84
Blood test	£5.21 (1.39 contacts)	£7.42	£0 to £26.18	£5.89 (1.58 contacts)	£6.68
X-ray	£12.05 (0.45 contacts)	£25.76	£0 to £106.08	£4.82 (0.18 contacts)	£13.99
Other outpatients	£73.38	£223.46	£0 to £ £979.20	£23.92	£75.31
Total cost of outpatient contacts	£1033.47	£756.30	£0 to £3455.76	£766.26	£596.74
Accident and Emergency	£78.76 (0.42 contacts)	£173.83	£0 to £742.56	£33.75	£86.26
Admissions					
Unplanned	£74.00 (0.030 nights)	£425.10	£0 to £2442	£891.48 (0.94 nights)	£4791.86
Planned	£88.36 (0.06 nights)	£353.28	£0 to £1458	£0.00 (0.00 nights)	–
Total cost of admissions	£162.36	£540.40	£0 to £2442	£891.48	£4791.86

	BGAT					
	Baseline to 12 months *n* = 25			12 to 24 months *n* = 32		
Min-Max	Mean	SD	Min-Max	Mean	SD	Min-Max
£0 to £200.50	£113.85 (2.96 contacts)	£126.30	£0 to £481.20	£72.25 (2.41 contacts)	£89.82	£0 to 336.66
£0 to £76.72	£23.97 (1.32 contacts)	£35.26	£0 to £172.62	£30.46 (1.63 contacts)	£69.49	£0 to £383.60
£0 to £842.40	£17.97 (0.32 contacts)	£57.82	£0 to £280.80	£23.09 (0.3 contacts)	£63.45	£0 to £336.96
£0 to £70.74	£11.32 (0.32 contacts)	£19.69	£0 to £70.74	£11.05 (0.31 contacts)	£18.93	£0 to £70.74
£0 to £16.80	£1.96 (0.28 contacts)	£3.42	£0 to £8.40	£1.22 (0.22 contacts)	£3.40	£0 to £13.96
_	£54.02 (1.80 contacts)	£253.83	£0 to £1270.92	£0.00 (0.00 contacts)	_	_
–	£0.83 (0.04 contacts)	£4.16	£0 to £20.78	£0.65 (0.031 contacts)	£3.67	£0 to £20.78
£0 to £242.30	£6.11 (0.12 contacts)	£25.62	£0 to £126.48	£14.66 (0.25 contacts)	£57.30	£0 to £316.20
–	£5.96 (0.08 contacts)	£20.62	£0 to £74.46	£0.00 (0.00 contacts)	–	–
£0 to £251.64	£40.26 (0.16 contacts)	£157.15	£0 to £754.92	£141.55 (0.56 contacts)	£711.57	£0 to £4026.24
–	£187.52 (1.84 contacts)	£669.69	£0 to £3290.21	£18.72 (0.19 contacts)	£59.13	£0 to £299.52
–	£78.87 (0.32 contacts)	£394.37	£0 to £1971.84	£7.70 (0.031 contacts)	£43.57	£0 to £246.48
–	£31.95 (0.32 contacts)	£124.70	£0 to £599.04	£3.12 (0.03 contacts)	£17.65	£0 to £99.84
–	£0.00 (0.00 contacts)	–	–	£0.00 (0.00 contacts)	–	–
£0 to £ 1591.20	£4.90 (0.16 contacts)	£24.48	£0 to £122.40	£0.00 (0.00 contacts)	–	–
–	£8.57 (0.28 contacts)	£42.84	£0 to £214.20	£0.00 (0.00 contacts)	–	–
£0 to £ 2225.86	£24.61	£96.04	£0 to £461.30	£0.00	–	–
£0 to £2355.49	£612.67	£960.82	£0 to £3732.63	£316.77	£732.94	£0 to £4152.61
£0 to £1884.96	£502.66 (3.2 contacts)	£384.77	£0 to £1256.64	£476.31 (3.03)	£664.62	£0 to £2827.44
£0 to £ 471.24	£6.28 (0.04 contacts)	£31.42	£0 to £157.08	£39.27	£169.27	£0 to £942.48
£0 to £628.32	£113.10 (0.72 contacts)	£96.40	£0 to £ 314.16	£68.72	£97.22	£0 to £314.16
£0 to £440.64	£30.84 (0.28 contacts)	£59.66	£0 to £220.32	£27.54	£48.46	£0 to £ 110.16
£0 to £ 183.60	£0.00 (0.00 contacts)	–	–	£8.61	£35.82	£0 to £ 183.60
£0 to £544.68	£43.57 (0.24 contacts)	£131.34	£0 to £544.68	£62.41	£209.36	£0 to £1089.36
£0 to £829.26	£99.51 (0.12 contacts)	£275.03	£0 to £ 829.26	£25.91	£146.59	£0 to £ 829.26
£0 to £ 29.92	£2.69 (0.72 contacts)	£4.11	£0 to £ 14.96	£3.04	£5.07	£0 to £ 22.44
£0 to £53.04	£4.24 (0.16 contacts)	£9.92	£0 to £26.52	£1.66	£6.52	£0 to £26.52
£0 to £ 373.32	£345.47	£1398.71	£0 to £7001.28	£21.77	£101.01	£0 to £564.67
£0 to £ 2273.24	£1148.38	£1509.23	£3.74 to £7849.92	£735.24	£802.51	£0 to £ 2834.92
£0 to £ 371.28	£44.56 (0.24 contacts)	£80.92	£0 to £185.64	£34.81	£87.42	£0 to £371.28
£0 to £27,521	£684.62 (0.83 nights)	£1878.96	£0 to £8320	£300.91	£817.89 (0.22 nights)	£0 to £2847
–	£59.44 (0.00 nights)	£297.20	£0 to £1486	£637.88 (0.44 nights)	£3608.37	£0 to £20,412
£0 to £27,521	£744.06	£1879.90	£0 to £8320	£938.78	£3645.96	£0 to £20,412

*Note*: Data from the AD-SUS.

Abbreviations: 2A, community services; 2B, secondary care services; *n*, number with data.

**TABLE 3:** Cost-effectiveness analysis.

HARPdoc vs. BGAT	0 to 12 months	12 to 24 months
Difference in mean total cost (adjusted)^a^	£525 (95% CI: −£832 to £1735)	-£719 (95% CI: −£2585 to £895)
Difference in mean QALYs (adjusted)^b^	0.029 (95% CI: −0.016 to 0.074)	0.037 (95% CI: −0.039 to 0.116)
Difference in mean total cost over 24 months (adjusted)	−£194 per participant (95% CI: −£2498 to £1942)	
Expected difference in QALYs over 24 months (adjusted)	0.067 per participant (95% CI: −0.024 to 0.155)	
Mean INB of HARPdoc vs. BGAT: base case *λ* = £20,000	£1521	Probability HARPdoc cost-effective (INB > 0) = 0.85
Mean INB: *λ* = £30,000	£2184	Probability HARPdoc cost-effective = 0.89
Mean INB: *λ* = £13,000	£1057	Probability HARPdoc cost-effective = 0.80

a 12-month model estimated on *N* = 58 observation. Expected value based on 1000 bootstrap replications. Baseline covariates used for adjustment: age, gender, method of insulin administration (trial stratification variable), presence of retinopathy, neurological symptoms, presence of cardiovascular disease, body mass index, duration of hypoglycaemic experience, previous attendance at ‘BERTIE’ course, previous attendance at other structured course. 24-month model estimated on *N* = 65 observations. Expected value based on 1000 bootstrap replicates. Baseline covariates: age, gender, method of insulin administration, use of insulin pump with automated suspended feature, body mass index.

b 12-month model estimated on *N* = 71 observation. Expected value based on 1000 bootstrap replicates. Baseline covariates: gender, method of insulin administration (trial stratification variable), EQ-5D-3L utility weight, duration of hypoglycaemic experience, duration of diabetes, previous attendance at other structured course, body mass index. 24-month model estimated on *N* = 61 observations. Expected value based on 1000 bootstrap replicates. Baseline covariates: gender, duration of hypoglycaemic experience, EQ-5D-3L utility weight, use of insulin pump with automated suspended feature.

## Data Availability

The data generated or analysed during this study are included in this published article or are available from the corresponding author on reasonable request.

## What’s new?

- A novel psychoeducational intervention called “HARPdoc”, targeting cognitions around hypoglycaemia in people with type 1 diabetes and treatment-resistant problematic hypoglycaemia, was shown to improve mental health and reduce problematic hypoglycaemia in a randomised controlled trial against a non-cognitive intervention.
- The present study demonstrates that HARPdoc, although more expensive to deliver, was cost-effective compared to its comparator, through off-target benefits on use of medical services and quality of life.
- HARPdoc is a valuable tool for implementation within the NHS as an adjunct to conventional therapies (education and technology) to minimise problematic hypoglycaemia and improve mental health and quality of life.
