# Supplementary material for: Cost-effectiveness of a Novel Hypoglycaemia Programme: The ‘HARPdoc vs BGAT’ RCT
Source: Diabet Med. Author manuscript; Available in PMC 2025 Dec 24. (PMC12728598; doi:10.1111/dme.15304)

Table S1: Form for documenting use of health services over preceding 12 months, collected at 12 and 24 months in the HARPdoc vs BGAT randomised controlled trial

Interviewer Name: ______________________

Enter 0 for services not used and do not double-count any service contacts within or between questions on this questionnaire. Do include services used because of Hypoglycaemia which are recorded on other questionnaires.

If the subject reports any contacts which are clearly part of the trial intervention (i.e. course contacts), please do not record them (we are measuring this separately and want to avoid double-counting).

1. In the last 12 months, have you had any planned or unplanned (emergency) hospital admissions that involved at least one night spent in hospital? *Include episodes of severe hypoglycaemia* NO

	Name of hospital	*Ward specialty*	*Prime cause of admission (write "planned or "emergency"*	*Number of nights (for day case procedures, please write ''**Day case**'')*
1st admission:				
2nd admission:				
3rd admission:				
Other admission:				
Other admission:				
Other admission:				

2. In the last 12 months, have you visited A&E, a hospital outpatient department or other hospital day service due to any illness? *Include episodes of severe hypoglycaemia* YES/NO

		Total number of visits in the last 12 months
Diabetes clinic *(including doctor / nurse / dietician) doc virtual, telephone clinic visit with doc lasted 5 min*		
Diabetes foot clinic		
Diabetes eye clinic screen		
Ophthalmology		
Blood tests (phlebotomy services)		
Dietetics		
General medical outpatients		
Day surgery centre		
A&E (record all visits including those resulting in admission to hospital)		
X-ray (not as part of other visits, but for x-ray only)		
Other, *please specify*:		
Other, *please specify*:		
Other, *please specify*:		

3. In the last <u>12 months</u>, have you had contact with any of the following?

	Total number of contacts in last 12 months							*Average duration of contact (minutes)*
		At surgery or clinic		*At home*		*By telephone*		
GP								
GP Practice nurse								

4. In the last <u>12 months</u>, have you had contact with any of the following?

To avoid "double-counting" this should <u>exclude</u> any hospital-based contacts recorded in section 2.

	Total number of contacts in last 12 months							*Average duration of contact (minutes)*
		At surgery or clinic		*At home*		*By telephone*		
NHS chiropodist/podiatrist								
NHS optician								
NHS district nurse								
NHS dietician								
NHS physiotherapist								
NHS occupational therapist								
NHS psychiatrist								
Privately paid for psychiatrist								
NHS psychologist								
Privately paid for psychologist								
NHS psychotherapist								
Privately paid for psychotherapist								
NHS counsellor								
Privately paid for counsellor								
Social worker								
Home help / care worker								
Meals on wheels								
Pharmacist for advice								
Other, *please specify*:								
Other, *please specify*:								
Other, *please specify*:								

Table S2: Unit costs for Health Services used for health economic analysis of HARPdoc vs BGAT in the HARPdoc RCT

Unit costs[1,2]

Community-based contacts	Cost per visit/contact
GP - at surgery or clinic	£40.10
GP - at home	£78.39
GP - by telephone	£15.86
GP Practice nurse - at surgery or clinic	£19.18
GP Practice nurse - at home	£30.26
GP Practice nurse - by telephone	£7.97
NHS chiropodist/podiatrist - at surgery or clinic	£56.16
NHS chiropodist/podiatrist - at home	£88.60
NHS chiropodist/podiatrist - at home	£23.34
NHS optician - at surgery or clinic	£35.37
NHS optician - at home	£55.80
NHS optician - by telephone	£14.70
NHS district nurse - at surgery or clinic	£19.18
NHS district nurse - at home	£30.26
NHS district nurse - by telephone	£7.97
NHS dietician - at surgery or clinic	£20.78
NHS dietician - total number of contacts in last 12 months at home	£32.78
NHS dietician - by telephone	£8.64
NHS physiotherapist - at surgery or clinic	£63.24
NHS physiotherapist - at home	£99.77
NHS physiotherapist -by telephone	£26.29
NHS occupational therapist - total number of contacts in at surgery or clinic	£48.96
NHS occupational therapist - at home	£77.24
NHS occupational therapist - by telephone	£20.35
NHS psychiatrist - at surgery or clinic	£251.64
NHS psychologist - at surgery or clinic	£99.84
NHS psychologist - at home	£195.17
NHS psychologist - by telephone	£41.50
NHS psychotherapist - at surgery or clinic	£246.48
NHS counsellor - at surgery or clinic	£99.84
NHS counsellor - at home	£157.52
NHS counsellor -by telephone	£41.50
Social worker - at home	£52.02
Home help / care worker - at home	£30.60
Meals on wheels - at home	£30.60
Pharmacist for advice - at surgery or clinic	£8.40
Pharmacist for advice - by telephone	£3.49
Health visitor	£75.60
Dentist (at dental practice)	£67.83
Midwife at home	£62.02
Breast feeding speclialist	£62.02
**Outpatient contacts**	
Diabetics clinic	£157.08
Opthalmology	£110.16
Dietetics	£91.80
Diabetic foot clinic	£157.08
Diabetic eye clinic	£157.08
Blood test (phelbotomy)	£3.74
X-Ray	£26.52
General outpatients	£181.56
AandE	£185.64
Day surgery	£829.26
**Other outpatient contacts**	
Cardiology	£144.84
Dentist	£174.42
Dermatology	£123.42
Antenatal diabetes	£291.72
Emergency eye treatment	£185.64
Gynaecology	£153.00
Hematology	£169.32
MRI	£25.63
Maternity unit diabetes	£291.72

Nephrology	£173.40
Neurology	£195.84
Psychology	£205.02
Pump DSN	£382.50
Pump DSN - phone calls and emails	£158.99
Rheumatology	£148.92
Rheumatology clinic	£148.92
Urgent care	£185.64
Ultrasound	£38.14
Gastroenterology	£147.90
OT	£74.46
Oncology	£196.86
Physio	£63.24
Allergy (ENT)	£114.24
Audiology	£116.28
Orthopaedic	£124.44
Pulmonology	£159.12
Endsoscopy and colonoscopy	£564.67
Urology	£112.20
Endocrinology	£164.22
**Inpatient admissions**	**Cost per bed day**
Planned long-stay	£1,458.00
Unplanned long-stay	£949.00
Unplanned short-stay	£2,442.00
Day case	£1,486.00
Psychiatric	£416.00

1. Data sourced from Jones, K. Burns, A (2021) Unit Costs of Health and Social Care, Personal Social Services Research Unit, University of Kent (various annual updates used) and National Health Service Reference Costs for 2017-2018.
2. All costs reported at 2020-21 prices levels with adjustments for input price inflation made using the Hospital and Community Services Index, the NHS Cost Inflation Index and the Personal Social Services Pay and Prices Index.

Table S3 **Calculating cost savings from reduced SH following HARPdoc and BGAT in the HARPdoc RCT, an additional analysis conducted for the discussion of the findings of the trial**

All costs taken from NHS reference costs 2020-21, (https://www.england.nhs.uk/wp-content/uploads/2023/04/2_National_schedule_of_NHS_costs_FY21-22_v3.xlsx).

To convert to US $, divide by 0.68.

Admissions¶ NES code	%	Cost in £ sterling	N at baseline (n = 91)	N 0 – 24 m (n = 85*)	
CC8+	44				
CC 5 – 7	27				
CC 3 – 4	17.45				
CC 0 – 2	10.5				
All admissions		570.85	7	2	
All ambulance call outs#			105	73	
Ambulance see and treat		268.39	67	54	
Ambulance see and convey		390.08	38	19	
A & E attendance costs+					
NES codes					
Cat 3 Investigation with Cat 1-3 Treatment	7.7%				
Cat 3 Investigation with Cat 1-3 Treatment	0.2%				
Cat 2 Investigation with Category 3 Treatment	1.6%				
Cat 2 Investigation with Category 3 Treatment	1.1%				
Cat 1 Investigation with Category 3-4 Treatment	2.0%				
Cat 1 Investigation with Category 3-4 Treatment	3.5%				
Cat 2 Investigation with Category 2 Treatment	9.1%				
cat 2 Investigation with Category 2 Treatment	10.7%				
Cat 2 Investigation with Category 1 Treatment	9.7%				
Cat 2 Investigation with Category 1 Treatment	11.4%				
Cat 1 Investigation with Category 1-2 Treatment	6.4%				
Cat 1 Investigation with Category 1-2 Treatment	36.6%				
A & E attendance costs+		117.3	38	19	

Assumptions:
* Number of patients providing data actual for baseline and estimated as mean of those providing data at 12 and 24 months for follow-up
¶ For admissions, we used weighted average cost of episodes, giving a cost per hospital admissions, £570.82

Ambulance call outs = see and treat and see and convey, not discriminated in patient questionnaire. Therefore we assumed see and treat = total ambulance events *minus* no of A&E attendences
Cost from = £268.39 for See and treat
Costs of see and convey added to A and E attendance costs. Each episode adds £390.08 to an A&E attendance
+ A&E attendences costed at *minimum* assuming *no* resus room utilisation and no Category 4 or 5 investigations and higher than category 3 treatment costs, even when associated with an admission
Notes: Also excluded are costs of glucagon used at home. All costs from UK data bases.
Cost savings were calculated as baseline costs *minus* 50% of costs incurred over 2 year follow up calculated per 100 patient years and then totalled for a minimum of 4% and a maximum of 10% of the adult T1 population falling into the category of people with treatment resistant problematic hypoglycaemia (https://www.diabetes.org.uk/professionals/position-statements-reports/statistics

Figure S1 **Cost-effectiveness acceptability curve (CEAC)[1] for base-case analysis,** showing the probability of HARPdoc being a more cost-effective alternative to BGAT at different cost-effectiveness thresholds. In the UK NICE currently adopt a threshold ranging from £20,000 to £30,000 per QALY gained. More recently a threshold of c.£13,000 per QALY gained has been put forward as a more appropriate value to adopt in NHS resource allocation decision making.

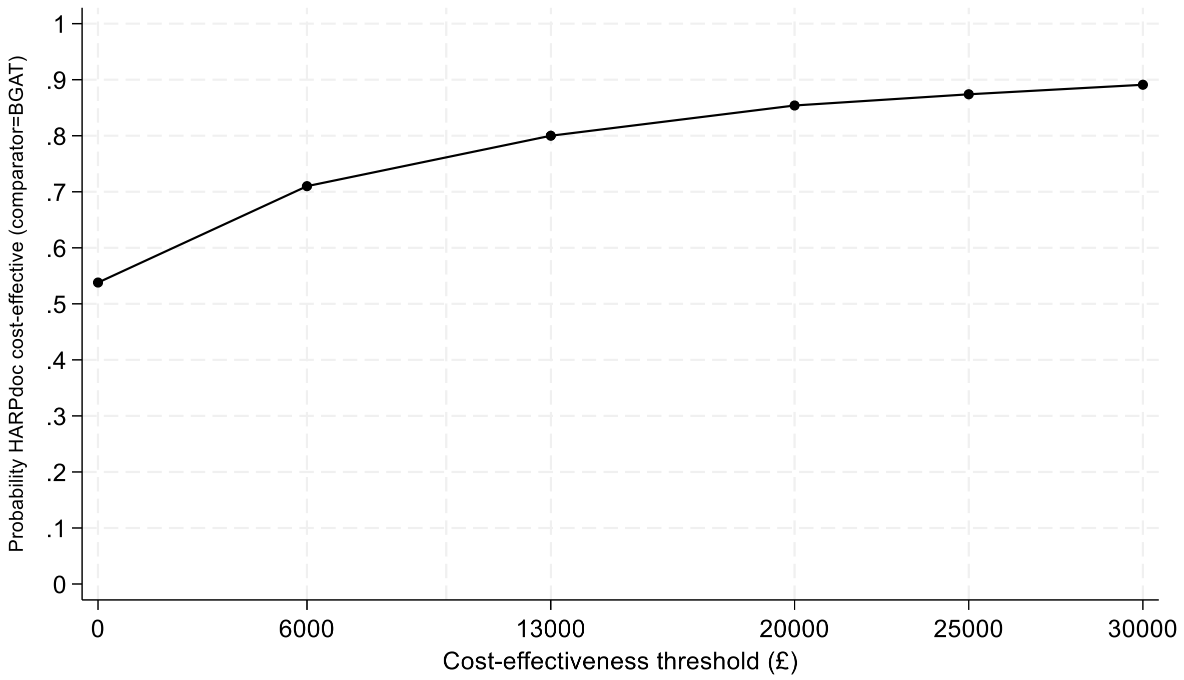

Supplement: supplemental material [file NIHMS2120731-supplement-supplemental_material.pdf]
